# A Text-Book of Human Physiology

**Published:** 1890-03

**Authors:** 


**A Text-Book of Human Physiology,** including Histology and Microscopical Anatomy; with special reference to the requirements of Practical Medicine. By Dr. L. LANDOIS, Professor of Physiology, University of Greifswald. Third American, translated from the Sixth German Edition, with additions by William Sterling, M. D., Sc. D., Brackenbury, Professor of Physiology and Histology in the Owens College, and Professor in the Victoria University, Manchester, etc. With 692 illustrations, 8vo, pp. 974. Philadelphia: P. Blakiston, Son & Co. Buffalo: Peter Paul & Bro. 1889.

This standard text-book is welcome in its third American edition. The translator has brought the science, of which he is an acknowledged exponent, well abreast of the period in this last rendering of the German into English, having made numerous additions to the author's text, with that end in view. These are enclosed in brackets so that the authorship is easily distinguished. The work now appears in a single volume, and the publishers have made it as handsome a specimen of the book-making art as can be produced.

Physiology is such a necessary complement to anatomy, and the two are so intimately blended, that it is impossible to acquire the one without some knowledge of the other. Moreover, it is as essential to the physician to constantly refresh his physiological studies as it is for the surgeon to keep himself intimately informed in all the anatomical minutiæ of the human body. Hence, it is important that both shall study these two corner-stones of the science of medicine with a sleepless, tireless energy, that shall keep them so amply equipped for the practice of their respective departments that no emergency can surprise them. This makes it imperative for all to examine with care all the latest and newest authorities in physiology and anatomy, and to compare the work of the several authorities with great accuracy. We believe that such a comparison will result in a favorable verdict for the work of Landois and Sterling, and, therefore, we unhesitatingly commend it to all who would do themselves even and exact justice. In the histological studies of these eminent authors will be found much of interest, as the latest authoritative expressions upon many moot points. The illustrations are especially to be commended as elucidating the text with great clearness, especially upon histology.

